# Comparative Nutritional and Physicochemical Profiling of Five Quinoa (*Chenopodium quinoa* Willd.) Varieties Cultivated in Burkina Faso

**DOI:** 10.3390/life16071064

**Published:** 2026-06-26

**Authors:** Elie W. W. Biego, Daniela I. Istrati, Camelia Vizireanu, Abdalla Dao, Oana E. Constantin, Eugenia M. Pricop, Maria C. Garnai, Philippe A. Nikiema, Hagrétou Sawadogo-Lingani

**Affiliations:** 1 Laboratory of Biological and Applied Sciences, New Dawn University, Bobo-Dioulasso 01 BP 234, Burkina Faso; biegoelie@yahoo.fr; 2 Faculty of Food Science and Engineering, Dunarea de Jos University of Galati, 111 Domneasca Street, 800201 Galati, Romania; camelia.vizireanu@ugal.ro (C.V.); emilia.constantin@ugal.ro (O.E.C.); mihaela.pricop@ugal.ro (E.M.P.); mgarnai@ugal.ro (M.C.G.); 3 Institute of Environment and Agricultural Research, Farako-Bâ, Bobo-Dioulasso BP910, Burkina Faso; dao_abdalla@yahoo.fr; 4 Department of Biochemistry Microbiology, Joseph KI-ZERBO University, Ouagadougou 03 BP 7021, Burkina Faso; pnikiema@gmail.com; 5 Department of Food Technology, Institute of Applied Science and Technology, Ouagadougou 03 BP 7047, Burkina Faso; hagretou@yahoo.fr

**Keywords:** Burkina Faso, quinoa (*Chenopodium quinoa* Willd.), nutritional characterization, amino acid profile, mineral composition

## Abstract

Since its introduction to Burkina Faso, quinoa (*Chenopodium quinoa* Willd.) has become a promising crop for diversifying cereal-based diets in a context marked by food insecurity, childhood malnutrition, and micronutrient deficiencies. This study compared five quinoa varieties (Titicaca, Psankalla, Puno, Negra Colana, and Salcedo Inia) cultivated in Burkina Faso, with emphasis on their relevance to human nutrition. Therefore, physicochemical parameters, proximate composition, amino acid profiles, and mineral composition were determined. Significant differences (*p* < 0.05) were observed among varieties for most parameters. Salcedo Inia exhibited the highest energy value (372.56 kcal/100 g), while Titicaca exhibited the greatest protein content (17.89 g/100 g) and lipid content (5.82 g/100 g). Psankalla and Puno presented favorable essential amino acid profiles, including lysine, supporting their potential use in cereal-based complementary foods. Negra Colana exhibited the highest crude fiber content (9.66 g/100 g) and the highest concentrations of several minerals, including iron, calcium, magnesium, and zinc, suggesting potential relevance for dietary strategies to improve micronutrient intake. The swelling behavior of selected quinoa varieties also suggests their suitability for soft, rehydrated formulations suitable for vulnerable populations. In general, Burkina Faso-cultivated quinoa varieties demonstrated promising nutritional attributes that could support dietary diversification, improve protein quality, and increase mineral intake. To our knowledge, this is the first study to systematically compare the nutritional profiles of five quinoa varieties cultivated under Sahelian agroecological conditions. Beyond its relevance for Burkina Faso, this study provides novel evidence on the expression of genotype-dependent nutritional traits in quinoa outside traditional production regions, supporting climate-resilient crop diversification and the development of quinoa-based complementary foods for vulnerable populations in food-insecure settings.

## 1. Introduction

Burkina Faso is facing significant issues in food security and nutrition, with 9.9% of children suffering from acute malnutrition, 19% stunted growth, and 13.2% underweight [1]. Micronutrient deficiencies are widespread, 41% are affected by anemia, 50.2% have vitamin A insufficiency, and 12.6% have zinc inadequacy. While progress has been made, with 70.8% of infants exclusively breastfed, 11.7% of the population (about 2.7 million individuals) remain food-insecure [1,2]. The primary factors contributing to food insecurity and malnutrition are insufficient minimum dietary diversity, substandard food quality, and the provision of inadequate supplementary items [1]. Also, the security situation has led to population relocation and the abandonment of agriculture and livelihoods. Sorghum, millet, and maize dominate in Burkina Faso [3]. It is therefore vital to identify methods to enhance and diversify the food supply. The increased production and use of quinoa (*Chenopodium quinoa*) are due to its environmental, agronomic, and health benefits [4]. This situation is not limited to Latin America, where the plant originated, but also affects the United States; Europe; Asia; and, most recently, Africa [4]. The plant under consideration has demonstrated an ability to thrive in a range of challenging agroclimatic conditions, including high altitudes, nutrient-poor soils, and temperature variations. It has been observed that maintaining productivity in arid soils or those with high salinity is difficult [5,6]. Some authors note that global quinoa output has risen over the past decade, surpassing 100,000 tons and sparking growing interest in its agricultural and nutritional potential. In West Africa, quinoa cultivation is still in its early stages. Since 2013, the Food and Agriculture Organization (FAO) has recognized this pseudocereal as one of the most promising crops for global food security. It has been identified as a potential solution to nutritional challenges. The FAO has been collaborating with various African countries to facilitate and promote its cultivation [7,8].

Quinoa is classified in the subphylum Tracheobionta, class Magnoliopsida, order Caryophyllales, family Amaranthaceae, and genus Chenopodium, which includes over 250 species worldwide. As a gluten-free pseudocereal, quinoa (*Chenopodium quinoa* Willd.) is increasingly recognized as a strong candidate for dietary diversification. It uniquely combines high-quality protein, an optimal balance of essential amino acids, dietary fiber, and significant mineral content [9]. Its small seeds vary in color from white to black, red, pink, and yellow, depending on the variety. The outer seed coat contains saponins, which are generally removed before consumption because they can impart bitterness and may influence nutritional acceptability and antinutritional load [4,9,10].

Quinoa stands out for its nutritional advantages over traditional grains such as millet, corn, rice, or sorghum. Its protein content, at 15.24–16.7% as reported [5,9,10,11], is higher than that of most grains. Quinoa proteins contain all essential amino acids, including lysine, which is often lacking in other grains. Studies show that quinoa has an in vitro protein digestibility (IVPD) of over 76%, a high essential amino acid index (EAAI ≈ 240%), and a high biological value (BV ≈ 251%), especially for black quinoa [9]. In addition, quinoa is naturally gluten-free [10], making it an excellent choice for individuals with celiac disease or other gluten-related digestive disorders [11]. The total fat content of these seeds ranges from 3.9% to 5.2% [12] with a fatty acid composition similar to that of corn, with a high proportion of polyunsaturated fatty acids, particularly linoleic acid (55–60%), α-linolenic acid (5–8%), and oleic acid (15–22%) [13]. The carbohydrate fraction of quinoa contains a significant amount of starch (58.1–64.2%) and dietary fiber (8.8–14.1%). The amount of fiber in quinoa grains is higher than that in wheat or rice and comparable to that in legumes [10,14,15,16].

Quinoa is a highly nutritious grain with a mineral content that exceeds that of local cereals such as millet, corn, and rice. Quinoa seeds contain magnesium (249.6 mg/100 g), phosphorus (383.7 mg/100 g), potassium (926.7 mg/100 g), iron (13.2 mg/100 g), zinc (4.4 mg/100 g), and calcium (148.7 mg/100 g) [17,18,19,20]. In addition to minerals, quinoa also provides important vitamins, such as vitamin B6 (487 µg/100 g) and folate (vitamin B9) (184 µg/100 g), sufficient amounts to cover the nutritional needs of children (70 µg/day for infants and 100 µg/day for children between 1 and 3 years). The riboflavin (vitamin B2) content, about 316 µg per 100 g, provides nearly 80% of the daily needs for children and almost 40% for adults [20,21].

Quinoa seeds contain numerous bioactive compounds, including saponins, polyphenols, flavonoids, phytosterols, and tocopherols [18,22]. These compounds contribute to quinoa’s antioxidant, anti-inflammatory, and metabolic-regulating properties. The total polyphenol content can reach up to 180 mg gallic acid equivalents (GAE) per 100 g of dry matter, and the level of phytosterols varies between 38 and 56 mg/100 g [18]. Additionally, α-tocopherol is the predominant form of vitamin E present in the seeds, further enhancing their functional potential.

Following its introduction in Burkina Faso in 2015 [7], quinoa has been the focus of research on agronomic aspects, and the results have confirmed that it is easily cultivated under Burkina Faso’s agroecological conditions [23]. According to the INERA (Institute of Environment and Agricultural Research) catalog, four varieties have been officially registered. However, quinoa remains virtually absent from local food consumption patterns, compared to other local cereals (corn, millet, and rice), which account for more than 90% of national cereal production and cover about 80% of arable land [24]. Quinoa faces persistent challenges related to low awareness among local consumers and agro-industry actors, which continue to limit its production and consumption in the country. To date, quinoa is not included in national food composition databases, household dietary surveys, or official agricultural production statistics, reflecting its negligible role in current food systems. However, its exceptional nutritional profile, particularly its balanced essential amino acid composition and high protein content, positions it as a promising ingredient for the formulation of nutrient-dense complementary foods targeting infant malnutrition. The lack of data on the nutritional characteristics of quinoa varieties grown under Sahelian conditions, therefore, represents a critical knowledge gap and directly justifies the present study. Consequently, to successfully integrate quinoa grains into various technological processes, it is essential to conduct a comprehensive study of their physicochemical, biochemical, and nutritional characteristics and assess their compatibility with these processes. No research has yet been conducted on the nutritional characteristics of the varieties introduced in Burkina Faso. This work aligns with international efforts such as FAO’s TCP/SFW/3404 (Phase I) and TCP/RAF/3602 (Phase II) project in West Africa, which aims to introduce and scale up the cultivation of the high-nutrient crop quinoa, and national strategies such as Alliance for a Green Revolution in Africa (AGRA Burkina), a 2023–2027 strategic plan that targets the adoption of nutrient-dense and climate-smart crop varieties by smallholder farmers.

Therefore, this study aimed to evaluate the nutritional quality of five quinoa varieties (Titicaca, Psankalla, Puno, Negra Colana, and Salcedo Inia) cultivated under Burkina Faso’s agro-climatic conditions and to highlight their potential contribution to human dietary quality. Particular attention was given to protein adequacy, essential amino acid supply, fiber intake, and mineral nutrition in populations exposed to malnutrition and food insecurity. By linking compositional data with practical nutrition priorities, this study aims to support the future selection of quinoa varieties for locally adapted, nutrition-sensitive food products.

## 2. Materials and Methods

### 2.1. Material

The plant material used in this study comprised five (05) varieties of quinoa (*Chenopodium quinoa* Willd.) obtained from producers in Soumousso, a locality located in the west of Burkina Faso (11°00′41″ north, 4°02′50″ west), in the Department of Karangasso-Vigué in the province of Houet, Guiriko Region of Burkina Faso. Therefore, the following varieties of quinoa (*Chenopodium quinoa* Willd.) were used in the present study: Titicaca, Psankalla, Puno, Negra Colana, and Salcedo. These cultivars have performed better in yield and technical itinerary control in earlier agronomic trials without any pesticide treatment. After harvesting, the grains underwent no chemical treatment for preservation; they were stored in a cold room (4 °C). Each variety’s 50 kg of seeds was cleaned and sun-dried. To preserve grains without chemicals, the samples were placed in double-hermetic “Pics” bags before examination. Two HDPE inside bags are within a woven polypropylene outer bag. They were then palletized in a dry area below 20 °C and 12% relative humidity. As varieties were cultivated under natural field conditions within the same agroecological zone, environmental variability was considered minimal. Still, it cannot be fully excluded as a potential source of variation in nutritional composition.

Before analysis, the seeds from all varieties were ground in an electric grinder for 3 min, and the resulting flour was passed through a laboratory sieve (500 µm mesh).

### 2.2. Methods

Determination of pH and titratable acidity 

The method employed in this study is similar to that of Nout et al. (1989) [25], with certain modifications. Approximately 10 g of flour was placed into a beaker, and then 20 milliliters of distilled water was added. The obtained mixture was homogenized using a magnetic stirrer (C-MAG HS 10, IKA Magnetic Stirrers, Staufen im Breisgau, Germany). The pH was then determined using a pH meter (Milwaukee MW102 PRO+, Milwaukee Instruments, Rocky Mount, NC, USA), which was calibrated with buffer solutions at pH 4 and 7.

To determine the titratable acidity, 50 mL of distilled water was added to the mixture used for measuring the pH, along with two to three drops of 1% phenolphthalein solution. After homogenization, the titratable acidity was determined by titration with 0.1 N NaOH until the first pink color persisted for at least 30 s. The analyses were performed in triplicate (*n* = 3). Titratable acidity is expressed as lactic acid equivalent (%) using the following formula:

(1) *Titratable acidity (%) = [90 × N × (V_T_/V_P_) × (V_NaOH_ × 0.001)]/PE × 100*

*N* = normality of NaOH (eq/L); *V*_T_ = total sample volume (mL); *V*_P_ = volume of test sample (mL); *V*_NaOH_ = volume of NaOH used during titration (mL); *PE* = test sample mass (g); and 90 = molar mass of lactic acid (g/mol).

Determination of the weight of 1000 grains

The 1000-grain weight was determined by the method described by ICRISAT (1997) [26]. For each variety, three separate batches of 1000 grains were manually enumerated, and the precise weight of each batch was subsequently determined by rigorous weighing on a laboratory analytical balance with a capacity of 200 g and an accuracy of 0.001 g (Kern ADB 200-4, Kern&Sohn GmbH Ziegelei 1, Balinger, Germany).

Determination of the swelling capacity

The determination of the swelling capacity was carried out by using the method described by Subramanian et al. (1986) [27]. The results were expressed as follows:

(2) *SC (%) = [(V_f_ − V_i_)/m] × 100*

*SC* = swelling capacity; *V*_i_ = initial volume of seeds or flour (mL); *V*_f_ = final volume after swelling (mL); and *m* = mass of the flour sample (g).

Water content determination

The water content was determined by measuring the weight loss after drying in an oven (Heratherm OGS60, Thermo Scientific, Langenselbold, Germany) at 105 ± 3 °C for 24 h, following the standard NF V03-707 (2000) [28]. The results are expressed as follows:

(3) *Water content (%) = [(Pe − (Pf − Po))/Pe]* × *100*

where:

Pe = weight of the sample before drying (g);

Pf = final weight of the dish plus dried sample (g);

Po = weight of the empty dish (g).

Ash content determination

The ash content was determined by calcination in a tubular oven (Nabertherm GmbH, Lilienthal, Germany) in accordance with the AOAC (2000) method [29]. The ash content was expressed as a percentage of 100 g of flour:

(4) *Ash content (%) = ((Pf − Po)/m) × 100*

where:

Po = the weight of the empty crucible (g);

Pf = the weight of the crucible containing the ash (g);

m = the mass of the sample (g).

Protein content determination

The protein content was quantified using the Kjeldahl method, also described by the AOAC (2000) [29]. The protein content was expressed as a percentage of 100 g of flour:

(5) *%N = ((Ve − Vb) × 0.1 × 0.14)/Pe*

where:

%N = nitrogen content (% *w*/*w*);

Ve = burette reading for the sample distillate (mL);

Vb = burette reading for the blank (mL);

0.1 = normality of the titrant used for titration of the distillate (eq/L);

Pe = sample mass (g);

0.14 = atomic mass of nitrogen (g·mol^−1^).

(6) *(%) Protein = %N × 6.25*

Fat content determination

The fat content of the samples was determined by Soxhlet extraction (Solvent Extractor SER 148/6, Vepl Scintifica, Usmate Velate, Italy), as described in the AOAC (2000) method [29]. The fat content is then calculated and expressed as a percentage of 100 g of flour:

(7) *%Fat = ((Pf − Po)/m)*

where:

%Fat = fat content on a fresh matter basis (%);

Po = weight of the empty extraction flask (g);

Pf = the weight of the flask containing the extracted fat (g);

m = the sample mass (g).

Crude Fiber Content Determination

The crude fiber content was determined according to AOAC Official Method 978.10 (2000) using a C. Gerhardt Fibretherm analyzer (Fibretherm FT12, C. Gerhardt GmbH & Co. KG, Königswinter, Germany), an automated system that simplifies traditional fiber analysis [30]. The crude fiber content is expressed as a percentage per 100 g of sample.

Carbohydrate content determination

The total carbohydrate content was determined by the difference method. The calculation is based on the values for protein, lipids, ash, crude fiber, and moisture. The carbohydrate content is expressed as a percentage of 100 g of flour:

(8) *% Carbohydrates content = 100 − (%Moisture + %Protein + %Fat + %Ash + %Fiber)*

The energy value determination

The energy value of the samples was calculated using the Atwater factors [31], according to which 1 g of carbohydrates provides 4 kcal, 1 g of protein provides 4 kcal, 1 g of lipids provides 9 kcal, and 1 g of fiber provides 2 kcal. Energy value was expressed in kcal/100 g and calculated using the following formula:

(9) *Energy (kcal/100 g) = (4 × %Protein) + (4 × %Carbohydrates) + (9 × %Fat) + (2 × %Fibers)*

Total amino acid profile

The total amino acid profile of the samples was determined by reversed-phase HPLC (Waters Corporation, Milford, MA, USA) using the Waters Pico-Tag method, as described by Bidlingmeyer et al. (1984) [32]. Amino acids were identified and quantified against external standards, and results are expressed as mg/100 g flour.

Analysis of mineral composition

The determination of iron, potassium, calcium, magnesium, sodium, and zinc contents was carried out by the AOAC 975.03 [33]. Flame atomic absorption spectrophotometry (FAAS) with an acetylene flame was used to quantify minerals at distinct wavelengths: 285.2 nm for magnesium, 248.3 nm for iron, 213.9 nm for zinc, 422.7 nm for calcium, 589.0 nm for sodium, and 766.5 nm for potassium. For each element, the proper standard solutions were used for calibration. Results are expressed in milligrams per 100 g.

Statistical analysis

The physicochemical and nutritional characteristics were expressed as mean values ± standard deviation across three replicates, using XLSTAT Pro 7.5.2. Before performing the ANOVA, the assumptions of normality and homogeneity of variances were assessed using the Shapiro–Wilk test and Levene’s test, respectively. When these assumptions were satisfied (*p* > 0.05), data were analyzed using a one-way ANOVA, and significant differences between means were identified using the Newman–Keuls post hoc test at *p* = 0.05.

## 3. Results

### 3.1. pH, Acidity, 1000-Grain Weight, and Swelling Capacity of Quinoa Seed Varieties

The results of pH, titratable acidity, and 1000-grain weight are recorded in Table 1.

The pH values of quinoa varieties ranged from 5.83 to 6.23. The Titicaca variety had the highest value, while the lowest was observed for the Negra Colana variety. The differences in pH between quinoa varieties reflect biochemical disparities that directly influence their technological behavior. The observed values are consistent with the 5.98 and 6.43 reported by [34]. The pH of quinoa is typically slightly acidic or neutral. The findings indicate that the Negra Colana variety has a lower pH of 5.83, which may indicate greater acidity and, consequently, affect its behavior during processing and the sensory profile of the final products. In contrast, the Titicaca variety has the highest pH, 6.23, approaching neutrality. It is widely accepted that a pH close to neutrality is conducive to the microbiological stability and technological quality of grains [35]. These pH values are broadly comparable to those reported for quinoa sourdough and malted quinoa systems, where titratable acidity values increase markedly upon fermentation [36], confirming that the raw, unfermented flours examined in the present study display the moderate, near-neutral acidity typical of unprocessed quinoa grain.

The titratable acidity ranged from 0.07 to 0.11%. The levels of different quinoa varieties are comparatively lower, a characteristic that is typical of cereals. The Psankalla variety, which exhibited the highest titratable acidity (0.11%), may indicate a higher organic acid content. Conversely, Negra Colana and Puno (0.07%) could be preferable in formulations where acidity is minimized. As demonstrated by Abderrahim et al. [37], elevated acidity levels can significantly influence the flavor, preservation, and fermentative properties of the grains. This variation may also indicate disparities in biochemical composition, particularly in organic acids and phenolic compounds [37].

Regarding the thousand-grain weight, values ranged from 1.12 g to 3.61 g. The Titicaca and Psankalla varieties had the highest weights, indicating that they tend to produce larger seeds. The lowest recorded value was that of the Negra Colana variety. The differences observed in thousand-grain weight between varieties are mainly due to genetic factors but may also be influenced by growing conditions and the biochemical composition of the grains. It can affect the selection of varieties based on processing needs [5]. These values are significantly lower than those of local cereals such as millet (12.1 g), corn (268.0 g), and sorghum (24.2 g), as reported by Songre et al. [38]. As a critical component of technological quality, this parameter has a substantial influence on milling yield, the texture of the resulting products, and their overall acceptability, as reported by Mu et al. and Gomez et al. [39,40]. Compared with quinoa grown in the Mediterranean zone of Chile, where thousand-grain weight ranged from 1.59 to 4.28 g across genotypes [41] and with Colombian quinoa accessions reporting comparable hundred-grain weight values [42], the range observed for the five Burkina Faso grown varieties falls within the lower portion of the values documented internationally, particularly for Negra Colana, suggesting that local agroecological conditions may constrain seed size relative to Andean or Mediterranean production environments.

The results of the swelling capacity of grains and flours of the five quinoa varieties, measured at regular time intervals (every 5 min), are presented in Table 2 and Table 3. The grains demonstrated a significantly higher swelling capacity than the flours.

The swelling capacity range for the grains was 200.43–281.25, demonstrating significant variation among the varieties. The Salcedo Inia variety shows the greatest swelling potential, reaching 281.50% at 55 min of soaking. The Negra collana variety shows a high initial swelling that subsequently slows down.

The swelling capacity of flour shows different trends, with average values ranging from 86.21% to 175.93%. Puno flour showed the highest swelling capacity after 30 min of soaking, while Negra Colana and Psankalla exhibited lower values throughout the soaking process. This parameter is a pivotal criterion for the use of quinoa in the food industry, significantly influencing the texture, palatability, and digestibility of finished products [39]. The swelling capacity of quinoa grains varies significantly over the 56 min. As demonstrated by the works of Mu et al. [39] and Triki et al. [43], this variability is contingent on the structure of starch, grain dimensions, and the contents of insoluble fiber and protein. The high swelling capacity of the Salcedo Inia variety can be attributed to its starch composition and low insoluble fiber content (Table 4), which promotes enhanced water absorption. This finding aligns with the conclusions of Belguet et al. [19] and Vilcacundo et al. [44] regarding the structuring effect of fibers. This variety is ideal for couscous preparation, as it has a consistent texture and rehydrates quickly. Milling changes grain structure, exposing carbohydrates and proteins to water. This may therefore explain the observed difference between grains and flours. Puno demonstrates technological suitability for applications requiring high swelling capacity (e.g., porridge, specialty breads, and extruded products). This trend is consistent with North-West European whole-meal flour trials, where Puno and Titicaca were likewise classified among the varieties with the strongest swelling behavior, displaying markedly higher water absorption indices than other tested genotypes [5]. Conversely, the comparatively low swelling capacity reported for whole quinoa seeds elsewhere (0.4 mL/g) [45] underscores that milling into flour, as performed in the present study, substantially enhances water absorption relative to intact grains, corroborating the grain-flour contrast observed here.

### 3.2. Chemical Composition and Nutritional Potential of the Five Quinoa Seed Varieties

This study examined the nutritional content of five different quinoa varieties and found clear differences in their chemical composition. The experimental data regarding the proximate composition and energy value of the samples are shown in Table 4.

The results show that the moisture contents are relatively low, at 9.56% and 10.55%, which is due to good post-harvest practices, particularly in packaging and storage. These values align with grain conservation standards, as reported by Pereira et al. [46] and Chen et al. [47], thereby ensuring good grain stability.

The protein content ranged from 10.27% to 17.89%. The highest protein values were presented by Titicaca, followed by Psankalla and Puno. Quinoa is recognized for a protein profile that is nutritionally attractive not only for its quantity but also for its essential amino acid balance, particularly its lysine content, which is often limited in cereal-based diets [4,13]. These differences in values could be linked to genetic, biochemical, and pedoclimatic factors. For the Puno, Titicaca, and Psankalla varieties, the values obtained for quinoa were higher than 14.2%, 13%, and 12.9%, respectively, as reported by Emine et al. [48], Mu et al. [39], and Vega-Galvez et al. [4]. The same observations apply to local cereals, as reported by Songre et al. [38], with millet (10.8%), maize (10.7%), and sorghum (10.8%) being the most prevalent. According to FAO (2002) [49], the recommended daily protein intake is 56 g/day for adult men and 46 g/day for adult women, and 10–14 g/day for infants (6–23 months). A 100 g serving of Titicaca (17.89 g/100 g) covers 31.9% of adult male and 127.8% of infant (12–23 months) protein requirements. Even Salcedo Inia (10.27 g/100 g), the lowest-protein variety, meets 18.3% of adult male and 73.4% of infant requirements per 100 g.

The lipid content ranged from 2.27% to 5.82%. The results on fat content show that Titicaca has a higher content of unsaturated fatty acids, confirming that quinoa is a relatively rich source of lipids. According to Vilcacundo et al. [44], Huang et al. [50], and Semmar et al. [20], the fat content of quinoa ranges from 2% to 9.5%. Our results were lower than those of Lilian et al., whose lipid content ranged from 5.5 to 7% [21]. According to Songre et al. [38], the lipid content of traditional cereals such as millet (6.1%) and maize (6.0%) was higher than that of quinoa [38]. Genetic and biochemical differences can explain these results. The FAO/WHO (2008) recommends that lipids comprise 20–35% of total energy for adults [51]. Among the five varieties, Titicaca (5.82 g/100 g) provides the highest lipid contribution (8.4% of adult daily requirements per 100 g), while Negra Colana (2.27 g/100 g) provides the lowest (3.3%). All five varieties serve as complementary lipid contributors in diversified diets. Taken together, the protein and lipid data suggest that selected quinoa varieties could improve the nutrient density of cereal-based diets, provided that their protein digestibility and antinutritional profile are also assessed.

The crude fiber content ranged from 1.21% to 9.66%. The crude fiber content measured in this study shows notable variation, with the Negra Colana variety exhibiting the highest value (9.66%), which closely aligns with the values reported by Navruz-Varli et al. [52] and Villacres et al. [53] (8.61%). A high-fiber diet has been shown to benefit the gastrointestinal system, thereby preventing common digestive disorders prevalent in communities with poor dietary habits, as reported by Repo-Carrasco et al. [54]. The WHO/FAO (2001) recommends 25–38 g of dietary fiber/day for adults [55]. A 100 g serving of Negra Colana covers 25–39% of adult daily fiber requirements, while Salcedo Inia covers only 3.2–4.8%, highlighting Negra Colana as the most relevant variety for fiber-related nutritional benefits in the Burkinabè context.

The carbohydrate content ranged from 57.92% to 71.20%. Carbohydrates represent the predominant component of quinoa varieties, which are considered a significant source of energy for children’s growth. The Salcedo Inia variety was distinguished from the other varieties by its carbohydrate content. Compared with local cereal varieties, the values for all varieties were lower than those of millet (74.1%), maize (73.7%), and sorghum (74.4%), as reported by Songre et al. [38]. According to FAO/WHO (2001), carbohydrates should contribute 45–65% of total daily energy intake [56]. A 100 g serving of Salcedo Inia (71.20 g/100 g) provides the highest carbohydrate contribution (17.5–25.3% of adult daily requirements), while Titicaca (57.92 g/100 g) provides the lowest (14.3–20.6%). Quinoa’s capacity to simultaneously supply energy and enhance functional performance in food formulations is demonstrated by its moderate carbohydrate and variable fiber content. Quinoa’s ability to provide energy, fiber, and higher-quality protein simultaneously supports its potential use in nutrient-dense food formulations, especially when combined with local ingredients to enhance acceptability and affordability.

The energy value indicates the amount of energy available from macronutrients. Salcedo Inia has been identified as a potential energy source due to its high apparent energy value, suggesting it may be a more suitable option for applications where energy is a priority. Conversely, Negra Colana, with a lower energy value, could be more ideal for low-calorie diets. The findings of this study are lower than those reported by Songre et al. [38] for millet, maize, and sorghum, at 393, 392.2, and 380.9 kcal, respectively, for local cereals. The macronutrient composition of quinoa directly affects its energy intake. FAO/WHO (2001) recommends 2500 kcal/day for adult men and 2000 kcal/day for adult women [56]. A 100 g serving of Salcedo Inia (372.56 kcal) covers 14.9% of adult male requirements, while Negra Colana (333.26 kcal) covers 13.3%. For complementary feeding, a 50 g serving of Salcedo Inia covers 93.1% of the 200 kcal/day target for infants 6–11 months, and a 100 g serving covers 67.7% of the 550 kcal/day target for infants 12–23 months.

The ash content ranged from 2.52% to 5.14%. The Negra Colana variety had the highest value. This may indicate a higher concentration of minerals. This mineral richness is consistent with the observations of Repo-Carrasco et al. [54], which highlight quinoa’s potential as a mineral source. Despite slightly lower energy values than traditional cereals, quinoa’s superior protein quality and mineral richness make it highly relevant for nutrition-enhancement initiatives.

In Table 5, the amino acid results showed significant variation (*p* < 0.05) among the five Burkina Faso-grown quinoa varieties, except for serine and histidine. The amino acid profiles confirm that quinoa is a complete protein source containing all essential amino acids, including lysine, which is typically the first limiting amino acid in cereal-based diets, consistent with the nutritional characterizations reported by Verena et al. [12], Vilcacundo et al. [44], and Villacrés et al. [53].

The Psankalla variety had the richest essential amino acid profile, with particularly high concentrations of isoleucine (752 µg/100 g), threonine (992 µg/100 g), leucine (714 µg/100 g), and lysine (1017 µg/100 g), thus confirming its superior nutritional potential, as reported by Chen et al. [47], and in agreement with the wide genotypic variability documented by Granado-Rodriguez et al. [57] and Reguera et al. [58]. The Puno variety’s distinctiveness is attributable to its exceptionally high proline content (6665 µg/100 g)—more than double that of any other variety—which could suggest increased resilience to environmental stresses, an advantage for cultivation in local semi-arid conditions [43] and consistent with the agroclimatic adaptations of quinoa varieties introduced in Burkina Faso [7,23]. The Titicaca variety had the lowest concentrations of most amino acids, notably methionine (123 µg/100 g), valine (209 µg/100 g), and phenylalanine (203 µg/100 g), which agrees with data reported by Reguera et al. [58] under contrasting agroecological conditions. The Negra Colana variety had the highest methionine content among the five varieties (846.0 µg/100 g). Methionine and cysteine constitute the first group of sulfur-containing amino acids, which are limiting most plant proteins, making this characteristic uniquely complementary for infant flour formulations [44,53]. The Negra Colana variety also had the highest alanine content (1657.8 µg/100 g), which could be associated with specific nitrogen metabolic pathways in dark-pigmented quinoa seeds [37]. However, its very low lysine concentration (154.7 µg/100 g)—the lowest of the five varieties—limits its use as the sole protein source in cereal-based infant diets [12]. The Salcedo Inia variety has the highest lysine content (2172.8 µg/100 g). It also exhibits very high levels of phenylalanine (936.8 mg/100 g DM) and methionine (512.8 µg/100 g DM). This result is consistent with the genotype-dependent nutritional variability documented by Reguera et al. [58] and Granado-Rodriguez et al. [57]. The results of this study confirm that no single variety has a uniformly optimal amino acid profile and that specific nutritional objectives can guide variety selection. In particular, the complementary profiles of Salcedo Inia (rich in lysine) and Negra Colana (rich in methionine) suggest that their combination in composite infant flour formulations could optimize the overall essential amino acid score, targeting the two most limiting amino acids in plant-based diets [44,53], with direct relevance to improving the nutritional quality of complementary infant foods in Burkina Faso [7,23].

As illustrated in Table 6, the mineral element composition (Fe, Ca, Zn, Mg, and P) of the five quinoa varieties is presented.

**Table 6:** Mineral composition (mg/g product) of quinoa varieties.

Quinoa Varieties	Fe	Ca	Zn	Mg	P
Negra Colana	142.30 ± 0.97 ^a^	350.04 ± 0.49 ^a^	5.08 ± 0.28 ^a^	321.77 ± 0.82 ^a^	326.83 ± 1.84 ^e^
Titicaca	21.44 ± 1.08 ^d^	100.29 ± 1.78 ^d^	4.68 ± 0.60 ^b^	242.90 ± 1.41 ^c^	423.41 ± 0.05 ^a^
Puno	18.61 ± 1.65 ^e^	225.55 ± 2.07 ^b^	3.22 ± 0.42 ^c^	302.33 ± 0.63 ^b^	418.32 ± 1.88 ^b^
Salcedo Inia	36.06 ± 0.84 ^c^	125.27 ± 1.51 ^c^	2.48 ± 0.31 ^d^	191.91 ± 1.34 ^e^	398.74 ± 1.99 ^c^
Psankalla	74.87± 1.35 ^b^	100.05 ± 0.42 ^d^	2.44 ± 1.24 ^d^	236.89 ± 1.26 ^d^	359.86 ± 0.59 ^d^
*p* Value	0.000	0.000	0.000	0.000	0.000

Means in the same column followed by the same lowercase letter do not differ significantly from each other (*p* < 0.05).

The mineral analysis results indicate that the quinoa varieties cultivated in Burkina Faso exhibit mineral levels either higher than or comparable to those reported in the literature [12]. Genotype, agroecological conditions, and soil–plant interactions strongly influence quinoa’s mineral profile. The values obtained for iron and calcium were high, but lower for the other parameters, compared to those reported by Ruiz et al. [8]. Compared to millet, maize, and sorghum, which have iron contents of 3.5, 1.9, and 3.5 mg/g, respectively, as reported by Songre et al. [38], the iron content of quinoa was significantly higher. The high iron content of Negra Colana identified in this study aligns with the antianemic properties reported for the closely related variety, Negra Collana, from the Peruvian Altiplano. In that case, extruded flour restored hemoglobin levels in anemic rats, with iron content reaching 19.8 mg/100 g [59].

Against FAO/WHO (2004) low bioavailability reference intakes (14.0–29.4 mg/day for adults; 18.6–27.4 mg/day for infants) [60,61], Negra Colana alone covers several-fold these requirements per 100 g serving, while even Puno, the lowest-iron variety, exceeds adult requirements. For calcium, magnesium, and phosphorus, the same high values relative to sorghum are observed as reported by Gerrano et al. [62]. Relative to FAO/WHO (2004) adult reference values (1000 mg Ca, 260 mg Mg, 700 mg P/day) [60], Negra Colana covers 35% of calcium and 124% of magnesium requirements per 100 g, the only variety exceeding 100% of adult magnesium needs, while Titicaca covers 60.5% of phosphorus requirements, the highest among all varieties. Finally, zinc concentrations were comparatively lower, ranging from 2.44 (Psankalla) to 5.08 mg/g (Negra Colana). For Salcedo Inia and Psankalla varieties, the values are consistent with those reported by Gerrano et al. [51] for sorghum (2.60 mg/g); conversely, Negra Colana, Titicaca, and Puno present higher values than sorghum. Against FAO/WHO (2004) zinc reference values adjusted for low dietary bioavailability [60,61], Negra Colana provides the highest contribution (46% of adult requirements per 100 g), whereas Psankalla and Salcedo Inia would require zinc-fortified complementation in infant food formulations. In this context, the distinctive attributes of the Negra Colana variety, which have iron and calcium levels 4 to 7 times higher than those of other varieties, could make it an essential crop for addressing major nutritional issues, such as reducing iron-deficiency anemia. Its significantly higher calcium content supports bone health and contributes to overall micronutrient intake.

**Figure 1:**
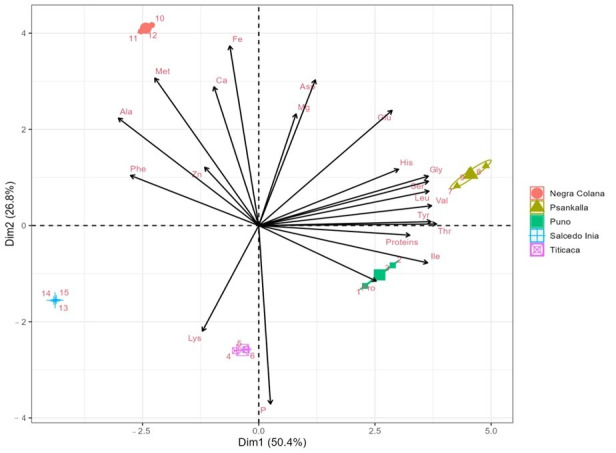
ACP biplot of quinoa varieties.

Principal component analysis (PCA) was performed on the integrated nutritional dataset that combined amino acid profiles, total protein content, and mineral composition across the five quinoa varieties. The resulting biplot (Figure 1), based on the first two principal components, accounted for 77.2% of the total variance, with PC1 and PC2 contributing 50.4% and 26.8%, respectively. This cumulative explained variance confirms an adequate two-dimensional representation of the overall nutritional variability among the varieties studied.

The first gradient (PC1, 50.4%) represents an amino acid richness dimension that primarily differentiates Psankalla and Puno from Negra Colana and Salcedo Inia. The positive association of Psankalla with the broadest range of essential amino acid vectors is consistent with the superior EAA composition reported in the amino acid profiling section and with the inter-varietal nutritional variability documented for quinoa grown under different agroecological conditions [57,58]. The tight alignment of Puno with the proline vector, together with its proximity to the total protein axis, reflects the osmotic stress response mechanism associated with proline accumulation in plants exposed to environmental constraints [43], which may constitute a specific adaptive trait favoring the cultivation of this variety under the semi-arid conditions of the Burkina Faso Sahel [7,23]. At the opposing end of PC1, the extreme isolation of Salcedo Inia is entirely driven by its lysine vector—a visual representation of the unique lysine accumulation pattern of this variety. Given that lysine is the primary limiting amino acid in cereal-based complementary foods in West Africa [12], this differentiation on the main variance axis positions Salcedo Inia as a nutritionally strategic ingredient for improving the protein quality score of infant flour formulations, in line with the framework proposed by Mariotti et al. for optimizing the essential amino acid adequacy of plant-based diets [63].

The second gradient (PC2, 26.8%) captures the mineral dimension of nutritional variability, with Negra Colana emerging as a clearly distinct entity along the positive PC2 axis. The co-directional loading of Fe, Ca, Mg, and Zn vectors toward the Negra Colana cluster reflects a coordinated mineral enrichment pattern that may be linked to the specific metabolic pathways associated with dark-seed pigmentation in this variety [37], as suggested by the simultaneous accumulation of Ala and Met in the same quadrant. The public health relevance of this mineral profile is considerable in the Burkina Faso context: iron deficiency anemia affects a substantial proportion of children under five in the Sahel, and zinc deficiency compromises immune function and growth during the critical window of complementary feeding, as extensively documented [64]. The opposing position of Titicaca along the negative PC2 axis, associated with the phosphorus vector, reflects its highest phosphorus concentration among all varieties—an element essential for ATP production, membrane integrity, and bone mineralization—and distinguishes its mineral profile from that of Negra Colana despite their shared negative PC1 scores.

Overall, the findings revealed apparent varietal differences in physicochemical, nutritional, and technological properties. These variations can be attributed to the combined effects of genotype, environmental conditions, and their interaction, as previously reported for quinoa by several authors [49,56]. The superior protein content and mineral composition highlight quinoa’s potential as a promising raw material for developing complementary foods tailored to local nutritional requirements.

### 3.3. Implications for Human Nutrition and Public Health

The five quinoa varieties produced in Burkina Faso collectively present a nutritional profile of direct relevance for addressing the multiple and concurrent burdens of malnutrition affecting the country, where acute malnutrition reaches 11.3%; chronic malnutrition reaches 35.1%; and iron deficiency anemia, zinc deficiency, and calcium insufficiency remain major public health challenges in children under five [55]. The protein contents of the five varieties (10.27–17.89 g/100 g), all significantly superior to conventional Sahelian cereals, confirm the nutritional positioning of quinoa as a high-value protein ingredient for complementary food formulations, consistent with the early work of Dao et al. [7,23] on the introduction and adaptation of quinoa in Burkina Faso. More importantly than protein quantity, the complete essential amino acid profiles documented here, particularly the exceptional lysine content of Salcedo Inia (2172.8 mg/100 g DM) and the balanced essential amino acid composition of Psankalla, address a critical nutritional gap in Sahelian diets, where lysine is the first limiting amino acid in cereal-based complementary foods consumed by infants from 6 months of age [12]. Mariotti and Gardner [63] demonstrated that plant protein digestibility is only marginally lower than animal protein (89–92% vs. 90–95%), confirming that quinoa-based formulations can provide effective protein nutrition without animal supplementation.

The mineral characterization reveals that Negra Colana stands out as an exceptional source of iron (142.30 mg/100 g), calcium (350.04 mg/100 g), magnesium (321.77 mg/100 g), and zinc (5.08 mg/100 g) concentrations that substantially exceed those of all Sahelian cereals and that are directly relevant for the prevention of the most prevalent micronutrient deficiencies in Burkina Faso. Razzaque and Wimalawansa [64] documented that iron deficiency affects more than 25% of the global population and is a major cause of anemia. At the same time, zinc, a cofactor for over 600 enzymes, is essential for immune function, growth, and cognitive development in young children. The high fiber content of Negra Colana (9.66 g/100 g) further contributes to intestinal health and glycemic regulation. The energy density of Titicaca (372.56 kcal/100 g) and its high lipid content (5.82 g/100 g), rich in essential fatty acids as documented for quinoa by Navruz-Varli and Sanlier [52], make it the most suitable variety for energy-dense infant gruels targeting children at risk of stunting, where adequate caloric intake in small food volumes is critical. These nutritional advantages are summarized in Table 4. The strategic pairing of Salcedo Inia (lysine-rich, highest EAA proportion) with Negra Colana (methionine-rich, highest mineral density) in composite infant flour formulations could simultaneously optimize the essential amino acid score and the micronutrient content, representing a locally sourced, climate-resilient solution to the nutritional challenges of complementary feeding in Burkina Faso and the broader Sahel region [7,23,44,53].

### 3.4. Limitations and Future Research Required for Health-Related Claims

Despite the comprehensive nutritional characterization provided by this study, several limitations must be acknowledged before the findings can be translated into health-related claims. First, the mineral concentrations reported reflect total content in raw grains, not biologically available fractions. Antinutritional factors, including phytic acid, saponins, polyphenols, and oxalates, are known to form insoluble complexes with iron, zinc, and calcium in the gastrointestinal tract, significantly reducing their effective absorption [44,64]. Since phytate and saponin contents were not determined in the present study, the degree to which the exceptional mineral concentrations of Negra Colana translate into nutritionally effective intakes remains to be established. Future studies should quantify antinutritional factors across all five varieties and conduct in vitro mineral bioaccessibility assays using the INFOGEST digestion protocol adapted for infant digestion, to provide a biologically meaningful picture of mineral availability.

Second, the protein quality assessment was based on total protein content and amino acid profiling after acid hydrolysis, without accounting for protein digestibility in gastrointestinal conditions. Protein digestibility is influenced by protein structure, processing conditions, and the presence of protease inhibitors [44,63] and cannot be inferred from amino acid composition alone. Future research should measure in vitro protein digestibility to fully validate the nutritional superiority of Salcedo Inia and Psankalla for infant nutrition.

## 4. Conclusions

The five quinoa varieties grown in Burkina Faso display considerable variation in their physicochemical, biochemical, and mineral traits, underscoring the crop’s adaptability and nutritional potential under local agroecological conditions. Higher mineral and fiber levels distinguish Negra Colana, whereas Titicaca presents higher lipid content, energy value, and grain swelling capacity. Psankalla provided a balanced protein profile enriched with essential amino acids, and Puno demonstrated a remarkable flour swelling capacity, accompanied by an elevated proline content, suggesting potential functional advantages. Salcedo Inia exhibited generally acceptable characteristics compared with the other varieties. As analyses were conducted on raw flour, reported values may not fully reflect the cooked products, since heat treatment generally reduces protein and mineral content through leaching while improving digestibility by reducing antinutrient levels. Ultimately, this study provides a foundation for developing optimized, high-nutrient food formulations tailored to local needs.

## Figures and Tables

**Table 1:** pH, titratable acidity, and 1000-grain weight of quinoa seed varieties.

Quinoa Varieties	pH	Acidity (%)	1000-Grain Weight (g)
Puno	6.20 ± 0.02 ^a^	0.07 ± 0.01 ^b^	2.39 ± 0.04 ^c^
Titicaca	6.23 ± 0.02 ^a^	0.09 ± 0.01 ^b^	3.61 ± 0.02 ^a^
Psankalla	6.0 ± 0.03 ^b^	0.11 ± 0.01 ^a^	3.04 ± 0.05 ^b^
Negra Colana	5.83 ± 0.02 ^c^	0.07 ± 0.00 ^b^	1.12 ± 0.01 ^e^
Salcedo Inia	6.01 ± 0.01 ^b^	0.08 ± 0.01 ^b^	2.11 ± 0.01 ^d^
*p* Value	0.0001	0.001	0.0001

Means in the same column followed by the same lowercase letter do not differ significantly from each other (*p* < 0.05).

**Table 2:** Swelling capacity of quinoa seeds.

Quinoa Varieties	Time (min)											
	5	10	15	20	25	30	35	40	45	50	55	60
Titicaca	11.76 ± 0.03 ^c^	23.53 ± 0.01 ^b^	29.41 ± 0.5 ^d^	47.06 ± 0.03 ^e^	82.35 ± 0.28 ^b^	123.53 ± 0.50 ^c^	152.94 ± 0.5 ^c^	176.47 ± 0.6 ^c^	200.00 ± 0.3 ^b^	217.65 ± 0.28 ^c^	235.29 ± 0.83 ^d^	241.18 ± 0.74 ^c^
Psankalla	6.72 ± 0.04 ^d^	13.725 ± 0.56 ^c^	26.89 ± 0.31 ^d^	53.50 ± 0.24 ^d^	74.04 ± 1 ^c^	93.54 ± 0.30 ^e^	120.00 ± 0.0 ^e^	147.01 ± 0.48 ^e^	167.17 ± 0.71 ^e^	180.13 ± 0.18 ^e^	193.79 ± 0.65 ^f^	200.43 ± 0.60 ^e^
Puno	18.24 ± 0.83 ^b^	24.43 ± 1.27 ^b^	35.63 ± 0.48 ^c^	59.09 ± 37 ^c^	82.51 ± 0.23 ^b^	135.06 ± 0.33 ^b^	157.88 ± 1.33 ^b^	183.51 ± 1.64 ^b^	195.45 ± 1.88 ^c^	252.10 ± 1.20 ^b^	258.82 ± 0.00 ^b^	258.82 ± 0.00 ^b^
Negra collana	28.02 ± 0.33 ^a^	60.675 ± 0.62 ^a^	67.71 ± 1.47 ^a^	73.52 ± 1.84 ^b^	82.72 ± 0.86 ^b^	112.22 ± 1.57 ^d^	134.40 ± 1.51 ^d^	155.34 ± 0.32 ^d^	177.30 ± 0.68 ^d^	194.85 ± 0.57 ^d^	216.67 ± 0.00 ^e^	216.67 ± 0.00 ^d^
Salcedo Inia	6.36 ± 0.16 ^d^	25.56 ± 0.79 ^b^	57.18 ± 1.32 ^b^	99.06 ± 1.34 ^a^	149.17 ± 1.18 ^a^	192.85 ± 0.3 ^a^	211.38 ± 1.59 ^a^	255.85 ± 0.57 ^a^	268.43 ± 0.45 ^a^	275.67 ± 0.94 ^a^	281.25 ± 0.00 ^a^	281.25 ± 0.00 ^a^
*p* Value	0.000	0.000	0.000	0.000	0.000	0.000	0.000	0.000	0.000	0.000	0.000	0.000

Means in the same column followed by the same lowercase letter do not differ significantly from each other (*p* < 0.05).

**Table 3:** Swelling capacity of quinoa flour.

Quinoa Varieties	Time (min)											
	5	10	15	20	25	30	35	40	45	50	55	60
Titicaca	10.00 ± 1.15 ^e^	15.00 ± 1.00 ^d^	42.00 ± 1.15 ^e^	55.00 ± 0.58 ^e^	70.00 ± 1.53 ^e^	85.00 ± 2.52 ^e^	98.00 ± 1.15 ^c^	98.00 ± 1.15 ^c^	98.00 ± 1.15 ^d^	98.00 ± 1.15 ^d^	98.00 ± 1.15 ^d^	98.00 ± 1.15 ^d^
Psankalla	31.91 ± 0.13 ^b^	51.00 ± 1.41 ^c^	62.82 ± 1.16 ^d^	77.64 ± 0.52 ^c^	90.09 ± 1.16 ^c^	109.54 ± 0.64 ^b^	114.32 ± 0.96 ^b^	126.64 ± 0.90 ^b^	135.68 ± 0.96 ^b^	135.68 ± 0.96 ^b^	135.68 ± 0.96 ^b^	135.68 ± 0.96 ^b^
Puno	11.17 ± 1.17 ^d^	68.99 ± 0.02 ^a^	76.18 ± 0.450 ^b^	103.73 ± 0.39 ^a^	119.85 ± 1.20 ^a^	136.96 ± 1.36 ^a^	152.36 ± 0.91 ^a^	175.93 ± 0.10 ^a^	175.93 ± 0.10 ^a^	175.93 ± 0.10 ^a^	175.93 ± 0.10 ^a^	175.93 ± 0.10 ^a^
Negra collana	27.46 ± 0.76 ^c^	62.01 ± 0.66 ^b^	84.81 ± 0.27 ^a^	92.80 ± 0.69 ^b^	101.34 ± 1.89 ^b^	104.43 ± 0.81 ^c^	111.77 ± 0.33 ^b^	124.04 ± 1.36 ^b^	124.04 ± 1.36 ^c^	124.04 ± 1.36 ^c^	124.04 ± 1.36 ^c^	124.04 ± 1.36 ^c^
Salcedo inia	37.97 ± 0.05 ^a^	62.54 ± 0.66 ^b^	69.55 ± 0.81 ^c^	75.27 ± 0.84 ^d^	79.97 ± 0.93 ^d^	83.38 ± 0.88 ^d^	87.11 ± 1.27 ^d^	87.11 ± 1.27 ^d^	87.11 ± 1.27 ^e^	87.11 ± 1.27 ^e^	87.11 ± 1.27 ^e^	87.11 ± 1.27 ^e^
*p* Value	0.000	0.000	0.000	0.000	0.000	0.000	0.000	0.000	0.000	0.000	0.000	0.000

Means in the same column followed by the same lowercase letter do not differ significantly from each other (*p* < 0.05).

**Table 4:** Proximate composition and energy value of five quinoa varieties produced in Burkina Faso.

Quinoa Varieties	Humidity (g/100 g)	Proteins (g/100 g)	Lipids (g/100 g)	Carbohydrates (g/100 g)	Crude Fiber (g/100 g)	Ashes (g/100 g)	Energy Value (Kcal/100 g)
Puno	10.23 ± 0.09 ^b^	17.73 ± 0.01 ^b^	4.71 ± 0.08 ^c^	59.14 ± 0.05 ^c^	4.85 ± 0.00 ^b^	3.34 ± 0.02 ^c^	359.59 ± 0.47 ^c^
Titicaca	10.00 ± 0.02 ^c^	17.89 ± 0.03 ^a^	5.82 ± 0.00 ^a^	57.92 ± 0.01 ^d^	5.28 ± 0.05 ^b^	3.09 ± 0.02 ^d^	366.16 ± 0.06 ^a^
Psankalla	10.55 ± 0.00 ^a^	17.80 ± 0.04 ^a^	5.00 ± 0.01 ^b^	60.19 ± 0.04 ^d^	2.60 ± 0.06 ^c^	4.87 ± 0.01 ^b^	362.12 ± 0.07 ^c^
Negra Colana	9.56 ± 0.03 ^d^	14.54 ± 0.03 ^c^	2.27 ± 0.01 ^d^	58.83 ± 0.03 ^b^	9.66 ± 0.11 ^a^	5.14 ± 0.01 ^a^	333.26 ± 0.10 ^d^
Salcedo Inia	9.89 ± 0.12 ^c^	10.27 ± 0.04 ^d^	4.92 ± 0.15 ^b^	71.20 ± 0.19 ^a^	1.21 ± 0.02 ^d^	2.52 ± 0.00 ^e^	372.56 ± 0.73 ^b^
*p* Value	0.0001	0.0001	0.0001	0.0001	0.0001	0.0001	0.0001

Means in the same column followed by the same lowercase letter do not differ significantly from each other (*p* < 0.05).

**Table 5:** Amino acid profile of the five varieties of quinoa produced in Burkina Faso (µg/100 g).

Quinoa Varieties	Asp	Glu	Ser	Gly	His *	Thr *	Ala	Pro	Tyr	Val *	Met *	Ile *	Leu *	Phe *	Lys *
Psankalla	938 ± 5.37 ^a^	1450 ± 5.65 ^a^	533 ± 2.19 ^a^	693 ± 1.55 ^a^	404 ± 3.32 ^a^	992 ± 2.54 ^a^	452 ± 2.19 ^a^	2745 ± 4.48 ^b^	386 ± 2.89 ^a^	462 ± 4.31 ^a^	280 ± 1.55 ^a^	752 ± 2.19 ^a^	714 ± 1.34 ^a^	415 ± 2.26 ^a^	1017 ± 2.89 ^a^
Puno	572 ± 0.71 ^b^	1307 ± 2.10 ^ab^	468 ± 6.22 ^a^	562 ±5.93 ^a^	202 ± 1.41 ^b^	695 ± 1.26 ^b^	357 ± 3.53 ^c^	6665 ± 2.05 ^a^	357 ± 2.19 ^a^	408 ± 2.89 ^a^	233 ± 4.66 ^ab^	53 ± 3.46 ^b^	509 ± 1.48 ^b^	281 ± 2.19 ^b^	793 ± 2.9 ^b^
Titicaca	482 ± 2.82 ^b^	826 ± 1.06 ^b^	303 ±2.82 ^b^	385 ± 1.97 ^b^	174 ± 6.36 ^b^	532 ± 0.70 ^b^	268 ± 3.13 ^b^	2369 ± 1.68 ^b^	214 ± 0.70 ^b^	209 ± 1.34 ^b^	123 ± 3.12 ^b^	545 ± 3.72 ^b^	432 ± 0.70 ^c^	203 ± 3.16 ^c^	745 ± 0.70 ^b^
Negra Colana	833.8 ± 1.12 ^c^	1191.3 ± 8.12 ^b^	339.7 ± 5.54 ^b^	428.3 ± 0.62 ^b^	192.2 ± 1.82 ^b^	362.3 ± 3.16 ^c^	1657.8 ± 2.84 ^a^	1206.8 ± 5.32 ^c^	195.5 ± 2.66 ^b^	206.3 ± 2.11 ^b^	846 ± 5.72 ^a^	231.7 ± 1.51 ^d^	391.5 ± 7.55 ^d^	650.7 ± 6.42 ^b^	154.7 ± 5.55 ^e^
Salcedo Inia	546.5 ± 4.45 ^d^	841.7 ± 1.2 ^b^	240 ± 8.13 ^c^	310.2 ± 9.62 ^c^	156 ± 0.13 ^c^	241.3 ± 1.55 ^d^	1392.2 ± 1.58 ^b^	1038.5 ± 0.73 ^c^	173 ± 1.13 ^b^	146.2 ± 1.81 ^c^	512.8 ± 1.64 ^b^	167.2 ± 2.02 ^d^	253.8 ± 5.91 ^e^	936.8 ± 8.63 ^a^	2172.8 ± 1.17 ^a^
*p* Value	0.001	0.034	0.274	0.006	0.158	0.046	0.000	0.000	0.007	0.008	0.023	0.004	0.001	0.004	0.003

Means in the same column followed by the same lowercase letter do not differ significantly from each other (*p* < 0.05). * Essential amino acid.

## Data Availability

The data supporting this study’s findings are available from the corresponding author (D.I.I.) upon reasonable request.
